# Longitudinal Effects of Stressful Life Events on Problematic Smartphone Use and the Mediating Roles of Mental Health Problems in Chinese Undergraduate Students

**DOI:** 10.3389/fpubh.2021.752210

**Published:** 2021-12-03

**Authors:** Chengjia Zhao, Nani Ding, Xue Yang, Huihui Xu, Xinyi Lai, Xiaolian Tu, Yijun Lv, Dongwu Xu, Guohua Zhang

**Affiliations:** ^1^School of Mental Health, Wenzhou Medical University, Wenzhou, China; ^2^School of Public Health and Management, Wenzhou Medical University, Wenzhou, China; ^3^Center for Health Behaviours Research, Faculty of Medicine, JC School of Public Health and Primary Care, The Chinese University of Hong Kong, Hong Kong, Hong Kong SAR, China; ^4^Renji College, Wenzhou Medical University, Wenzhou, China; ^5^Wenzhou Medical University, Wenzhou, China; ^6^The Affiliated Kangning Hospital, Wenzhou Medical University, Wenzhou, China; ^7^Key Laboratory of Alzheimer's Disease of Zhejiang (P)rovince, Institute of Aging, Wenzhou Medical University, Wenzhou, China

**Keywords:** stressful life events, depressive symptoms, sleep quality, suicidal ideation, longitudinal study, problematic smartphone use

## Abstract

**Background and Aims:** This three-wave longitudinal study investigated the effects of stressful life events on problematic smartphone use and the mediating roles of mental health problems (i.e., depressive symptoms, poor sleep quality, and suicidal ideation) in Chinese undergraduate students.

**Methods:** A total of 197 undergraduate students completed the three-wave surveys. Their severity of stressful life events, mental health problems, and problematic smartphone use were assessed.

**Results:** Regression analyses revealed that stressful life events at T1 was significantly associated with problematic smartphone use at T3. Mediation analyses showed that mental health problems (i.e., depressive symptoms, poor sleep quality, and suicidal ideation) at T2 fully mediated the association between stressful life events at T1 and problematic smartphone use at T3 (B = 0.042, 0.034, and 0.022, respectively).

**Conclusions:** The present study revealed that stressful life events and mental health problems (i.e., depressive symptoms, poor sleep quality, and suicidal ideation) are predictors of problematic smartphone use in Chinese college students.

## Introduction

Mobile media use has become a necessity of life around the world. Mobile phones expedite communication without restrictions due to physical contiguity or spatial fixation and empower users to perform a variety of online activities, like online e-meeting, online gaming, and other online services (1–3). In the past decade, the frequency of smartphone use among young people in Asia and Europe has increased dramatically (4). For example, as of July 2020, there were about 932 million smartphone users in China, of which 23.7 percent were students (5). College students are generally highly motivated to use smartphones and update them quickly, and they are also the fastest adopters and users of new applications and new programs (6). Recent researches show that college students generally own smartphones (7, 8), excessive use of a smartphone can lead to problematic smartphone use (9, 10).

Problematic smartphone use (PSU) refers to the excessive use of the smartphone or smartphone addiction in daily life, accompanied by dysfunction and symptoms similar to substance use disorder (11). PSU severity is associated with a wide variety of indicators, such as stress (10, 12, 13), symptoms of depression (12, 14, 15), poor sleep quality (15, 16), and suicidal ideation (17). However, most of this research is based on cross-sectional studies, with only a few longitudinal studies examining risk or protective factors predicting PSU symptoms over time. Given the negative consequences of PSU, it is crucial to explore the possible psychological mechanisms of PSU symptoms utilizing longitudinal designs.

Stressful life events refer to the things that compel people to make changes in their ongoing life patterns (18), such as study pressure, and interpersonal tension (19). Stress has been well-documented as a risk element in the development of addiction and the vulnerability to relapse (10, 12, 20), and stressful life events have also been considered important factors potentially contributing to the development of PSU (13). Some authors conceptualize PSU as dysfunctional coping with everyday life (10, 13). Smartphone use has become a convenient and popular means for stress relief, entertainment, and social connection in young people (8). Empirical studies revealed that stressful life events were positively correlated with problematic or addictive online behaviors, such as online gaming addiction, Internet addiction, and problematic smartphone use (13, 21, 22). Longitudinal studies have also demonstrated that problematic smartphone use can be predicted by stressful life events (13, 23).

Undergraduate students are at an important transitional stage and may face various new challenges, such as adjustment to college life, poverty, academic pressures, part-time work, and identity changes (24–26). These stressors might increase the risk of stress-induced mental health problems in undergraduate students, such as depression (27), poor sleep quality (28, 29), and suicidal ideation (30). There is good evidence that one of the most important risk factors for mental health problems is stressful life events (31, 32).

According to the Diathesis Stress Model of Depression, depression may be the result of the combined effect of stress and individual susceptibility to diathesis (33). Several studies have confirmed that there was a positive correlation between stressful life events and depression (34, 35), stressful life events is a risk factor for depression (36), and have a direct impact on individuals' depressive symptoms (37, 38). Longitudinal findings have also revealed that stressful life events can predict depression symptoms over time (13, 39).

Stressful life events may also reduce poor sleep quality. Sterling and Eyer (40) put forward the concept of the “unsteady state,” whereby an organism's stress response is the process of reaching a steady-state again. However, in the case of severe and prolonged stress, this process will gradually become disordered and collapse, leading to an “unsteady state.” Stress triggers the body's physical and mental responses, and these regulatory effects can affect sleep quality. Previous studies have demonstrated the positive association between stressful life events and poor sleep quality, including both cross-sectional studies (41) and longitudinal studies (28, 29).

Suicidal ideation refers to thoughts about suicide and serious self-injury behaviors; it is the pre-clinical stage of suicide and can predict suicide behaviors (42). The stress theory of suicide proposes that long-term stress or coping failure can also cause suicidal ideation or behavior; stressful life events are the most common stressors of suicidal ideation (43–45). People with suicidal ideation have often experienced major stressful, negative, or traumatic sexual events (46), especially in adolescents (44). Longitudinal research has also shown that stressful life events significantly predicted suicidal ideation (30).

Nowadays, the smartphone's small size and portability enable persons to constantly access online (and offline) content, potentially causing overuse (47). Individuals who experience affective mental health problems (such as depression, poor sleep quality, and suicidal ideation) are more likely to increase smartphone use in daily life (48, 49). Therefore, mental health problems are considered as the influential risk factors for PSU (11, 49, 50). Associations between mental health problems and PSU have been evaluated through several studies. For instance, a systematic review and meta-analysis has found that mental health problems were significantly linked to PSU (51). Some studies have also suggested that PSU can be predicted by depression (11, 14), sleep problems (50), and suicidal ideation (49).

### Theoretical Framework

The Interaction of Person-Affect-Cognition-Execution (I-PACE) model (52) presents a comprehensive theoretical framework that attempts to explain the mechanism of Internet-related overuse. According to the I-PACE model, predisposing characteristics are important for PSU, such as personality, mental health (e.g., anxiety and depression), genetics, and biology in general; Cognitive and affective consequences are important influences, such as coping styles, executive impairment, mood dysregulation, and internet-related cognitive bias. The I-PACE model assumes that these cognitive and affective consequences involve responses to predisposing characteristics and can lead to healthy enjoyment through technology or excessive use, including PSU (9). Empirical studies suggest that mental health problems such as depressive symptoms, sleep quality and suicidal ideation are products of stressful life events (28–30), and they can lead to PSU (11, 49, 50). Based on the I-PACE model (52) and existing literature, mental health problems (i.e., depressive symptoms, sleep quality, and suicidal ideation) may play mediating roles in the association between stressful life events and PSU.

### The Present Study

**Figure 1:**
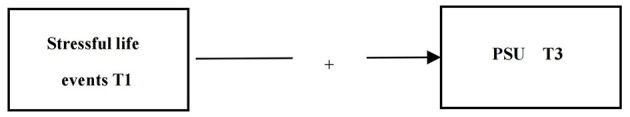
Visualization of Hypothesis 1.

**Figure 2:**
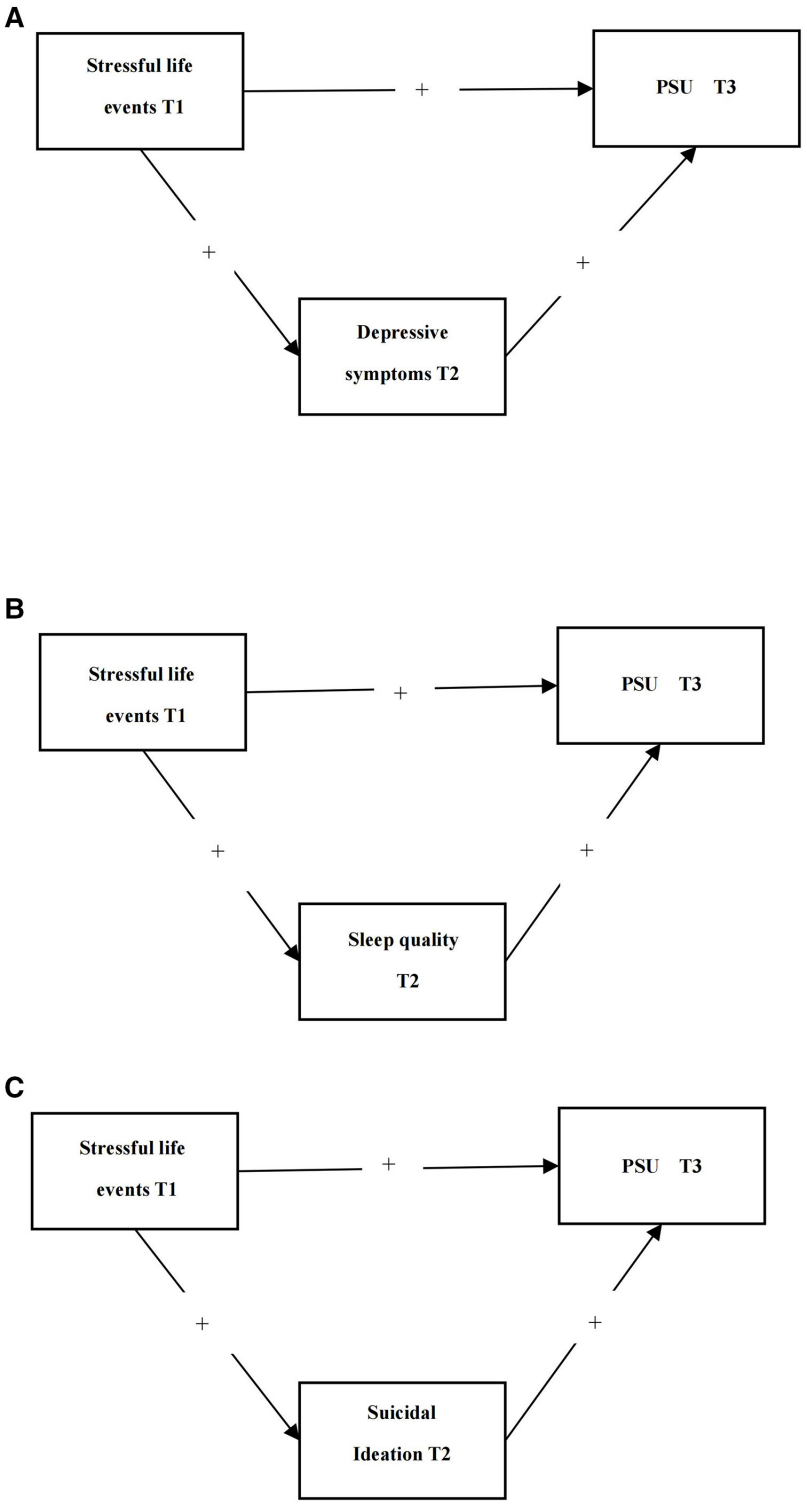
Visualization of Hypothesis 2: **(A)** Hypothesis 2a; **(B)** Hypothesis 2b; and **(C)** Hypothesis 2c.

The three-wave longitudinal surveys of Chinese undergraduates were used in this study. We hypothesized that stressful life events at T1 would positively predict PSU at T3 (Hypothesis 1). Figure 1 presents Hypotheses 1. Furthermore, we hypothesized that depressive symptoms at T2 would mediate the positive relationships between stressful life events at T1 and PSU at T3 (Hypothesis 2a), and also proposes sleep quality at T2 would mediate the relationship between stressful life events at T1 and PSU at T3 (Hypothesis 2b), as well as suicidal ideation at T2 would mediate the relationships between stressful life events at T1 and PSU at T3 (Hypothesis 2c). Figure 2 presents Hypotheses 2a, 2b, and 2c.

## Materials and Methods

### Participants

Undergraduate students from Wenzhou Medical University in Wenzhou city, Zhejiang Province, China were the study samples. The inclusion criteria of this study were as follows: (1) willingness to participate in the baseline and follow-up surveys; and (2) having used a smartphone on an everyday basis in the past year. The study invited 219 students in four majors (i.e., Forensic Medicine, Stomatology, Anesthesia Medicine, and Traditional Chinese Medicine) to participate in the questionnaire surveys. A total of 212, 205, and 197 students completed the survey at baseline (T1; response rate: 96.8%), T2, and T3, respectively; 197 students completed all three waves of the survey. Of these 197 participants, 58.9% were female, 52.3% were from urban areas, 48.5% lived in the one-child family, and 58.6% spent more than 4 h a day on smartphones.

### Materials

#### Adolescent Self-Rating Life Events Checklist

The 27-item Adolescent Self-Rating Life Events Checklist (19) was used to evaluate the intensity and frequency of stressful life events in undergraduate students in the past year. It is divided into six dimensions: learning pressure (e.g., heavy study burden), interpersonal relationship (e.g., discriminated against or ignored), punishment (e.g., criticized or punished), loss (e.g., friends and relatives died), health adaptation (e.g., being away from family for a long time), and other (e.g., a bad relationship or failure in love). Participants were told to recall whether such events had occurred during the preceding 12 months of their lives. If they answered “no, the score was 0; when answering “yes,” they were required to assess the impact of the stressful life event from 1 (not at all) to 5 (very much), with higher sum scores (0–135) representing more experiences of stressful life events. The good reliability and validity of this scale have been shown in previous studies (53, 54), as well as in the current study (Cronbach's α T1 = 0.91, α T2 = 0.94, α T3 = 0.94).

#### Mobile Phone Addiction Index Scale

The Mobile Phone Addiction Index Scale (55) contains 17 questions, get adapted to measure the PSU. Eight items of Young's Internet Addiction Diagnosis Questionnaire (56) were adapted to form eight items (i.e., items 3, 5, 6, 7, 8, 10, 15, and 16) of this scale. An example item is “When you are feeling down, you play with your smartphone to improve your mood.” Items are rated on a Likert scale ranging from 1 (never) to 5 (always), with higher sum scores representing higher levels of excessive smartphone use. The scale has been found to have good reliability and validity and to be suitable for assessing the excessive use of smartphones in Chinese populations (57). Reliability in the current study was good (Cronbach's α T1 = 0.85, α T2 = 0.88, α T3 = 0.91).

#### Center for Epidemiologic Studies Depression Scale

Depressive symptoms were assessed using the Chinese version of the 20-item Center for Epidemiologic Studies Depression (CESD) Scale (58) at T1, T2, and T3. The scale assesses the current severity of depressive symptoms, emphasis is placed on the emotional component of depression (59). The CESD can be used to indicate possible depressive symptoms, and its outcome score is significantly associated with clinical assessment results (59, 60). Furthermore, possible depressive symptoms has been found to predict depression diagnosis (61). Over the past 7 days, participants scored on a 4-point scale from 0 (little or none) to 3 (almost all of all) on how often they experienced symptoms such as restless sleep and feeling lonely. Reliability in the current sample was good (Cronbach's α T1 = 0.90, α T2 = 0.92, α T3 = 0.89).

#### Pittsburgh Sleep Quality Index

The 18-item Pittsburgh Sleep Quality Index revised by Liu (62) was used to evaluate the sleep quality of the subjects for nearly 1 month. The scale measures the following seven factors, each factor is scored on a scale from 0 to 3, and the total score of all seven factors is the total score of the scale. The higher the score, the poorer the quality of sleep. An example item is “Have you had enough energy to do anything in the past month?” Because this scale has good reliability and validity, it is used to measure the sleep quality of Chinese populations (63). Reliability in this current study was acceptable (Cronbach's α T1 = 0.68, α T2 = 0.71, α T3 = 0.69).

#### Self-Rating Idea of Suicide Scale

The 26-item Self-Rating Idea of Suicide Scale was used to evaluate the suicidal ideation of undergraduate students (64). The 26 questions are used to assess the four following factors: despair (12 questions), optimism (4 questions), sleep (4 questions), and concealment (5 questions). Each item is scored as “yes” or “no”; For positive items, the score is positive, with a “yes” score of 1 and a “no” score 0, whereas for negative items, the score is reversed, with a “no” score 1 and a “yes” score 0. An example item is “I often feel pessimistic and disappointed.” The scale has good reliability and validity and is suitable for measuring suicidal ideation in the Chinese population (65). Individuals with high levels of suicidal ideation scored high. If the masking factor (sometimes I also gossip about other people) score was ≥4, which indicates that the subject was unwilling, to tell the truth, the data were removed from the study. Reliability in this study was acceptable (Cronbach's α T1 = 0.67, α T2 = 0.72, α T3 = 0.71).

### Procedure

The three-wave surveys were conducted at the end of each semester (December 2018/June 2019/January 2020) in the first year and the second year of college. All surveys were conducted in classroom settings. A well-trained and experienced research assistant explained to participants that their participation was voluntary, and refusal to participate would not result in any negative consequences. Data confidentiality was guaranteed and only the researchers could access the data. Student IDs were collected for data matching. Researchers were not able to access students' names or other identifying information. Participants who completed the study were paid 60 RMB (9.27 dollars).

### Statistical Analyses

Statistical analyses were conducted with the Statistical Package for the Social Sciences 24 (SPSS, New York, NY, USA) and the PROCESS macro version 3.3 (www. processmacro.org/index.html) which the Process macro from SPSS. All variables of interest were normally distributed; the skewness and kurtosis of stressful life events, PSU and mental health problems (i.e., depressive symptoms, sleep quality, and suicidal ideation), fell within the acceptable range [i.e., skewness < |2.0| and kurtosis < |7.0|; (66)]. First, repeated-measures analyses of variance (within-subject ANOVAs) were applied to assess changes of the studied variables between T1, T2, and T3. Then, the correlations between all variables were investigated using zero-order bivariate correlations. Next, two-step hierarchical regression analyses (95% confidence intervals [CIs]) were performed to test the associations between independent variables and dependent variables. In the regression model, sex was included as a control variable in Step 1. In Step 2, stressful life events at T1 was entered as an independent variable, and PSU at T3 were entered as dependent variables (see Hypotheses 1). Finally, the mediation effects of mental health problems (i.e., depressive symptoms were tested using bootstrapping analyses (5,000 resamples) via Process (model 4; see Hypotheses 2a, 2b, and 2c) and 95% CIs are reported. Effect sizes (*PM* = the ratio of the indirect effect to the total effect) are also reported (67). Given the multiple testing, *p*-values were corrected by applying the Holm correction [level of significance: *p* < 0.05; (68)]. A priori power analyses (G^*^Power program, version 3.1) indicated that a sample size of at least *N* = 86 was required for valid results [Power >0.80, α = 0.05; (69)].

## Results

We compared the characteristics of those who were followed up (*n* = 197) vs. those who were missing in the first and third follow-up surveys (*n* = 15). The two groups did not differ in socio-demographic characteristics (sex, age, and major) or the levels of the independent and dependent variables (*p* > 0.05).

As shown in Table 1, the levels of stressful life events, PSU, sleep quality, and suicidal ideation were significantly different. Namely, stressful life events and PSU were significantly lower at T2 and T3 than that at T1 (T2/T3 < T1) (*F* = 10.176, *p* < 0.05; *F* = 8.062, *p* < 0.001). Sleep quality and suicidal ideation were significantly lower at T1 and T2 than that at T3 (T1/T2 < T3) (*F* = 88.370, *p* < 0.001; *F* = 296.026, *p* < 0.001). The levels of depressive symptoms at T1, T2, and T3 were not significantly different (*F* = 0.063, *p* = 0.802).

**Table 1:** Mean, SD, minimum, maximum, and repeated-measures ANOVAs (T1, T2, and T3) of all variables.

	**T1**		**T2**		**T3**		
	**M (SD)**	**Min–Max**	**M (SD)**	**Min–Max**	**M (SD)**	**Min–Max**	***F***	***p***	**Partial η^2^**
Stressful life	30.05 (17.51)	0–103	25.46 (18.77)	0–98	25.84 (18.93)	0–102	10.176	<0.05	0.05
Events									
Depressive symptoms	14.63 (8.32)	0–41	15.36 (9.27)	0–43	15.87 (9.94)	0–50	0.063	0.802	0.00
Sleep quality	5.54 (1.98)	1–12	5.66 (2.40)	1–16	8.62 (2.49)	4–18	88.370	<0.001	0.31
Suicidal ideation	3.87 (2.78)	0–16	3.97 (2.87)	0–14	5.89 (3.32)	0–16	296.026	<0.001	0.60
PSU	48.27 (10.57)	17–74	44.72 (11.27)	16–73	46.06 (12.66)	17–85	8.062	<0.05	0.04

*N, 197; PSU, problematic smartphone use; M, mean; SD, standard deviation; Min, minimum; Max, maximum; effect size, Partial η^2^*.

Table 2 shows the correlations between the investigated variables at T1, T2, and T3. Stressful life events at T1 were significantly and positively associated with depressive symptoms, sleep quality, and suicidal ideation at T2, as well as PSU at T3.

**Table 2:** Correlations between variables at T1, T2, and T3.

	**1**	**2**	**3**	**4**	**5**
1. SLE T1	1	0.14^*^	0.28^***^	0.14^*^	0.17^*^
2. DST2		1	0.54^***^	0.53^***^	0.31^***^
3. SQT2			1	0.37^***^	0.26^***^
4. SIT2				1	0.39^***^
5. PSU T3					1

*N = 197. SLE, stressful life events; DS, depressive symptoms; SQ, sleep quality; SI, suicidal ideation; PSU, problematic smartphone use*.

*** *p < 0.001 and*

* *p < 0.05*.

Regression analyses (Table 3) revealed that stressful life events at T1 was a significant predictor of the PSU at T3. These analyses revealed a predictive variance of 2.8% for PSU at T3.

**Table 3:** Hierarchical regression analyses predicting PSU at T3.

	**β**	**[95% CI]**	***T***	**Adjusted *R*^2^**	**Changes in *R*^2^**
Step 1, *F*_(1, 195)_ = 8.895, *p* < 0.01				0.007	
Sex	−0.112	[−0.511, 0.058]	−1.567		
Step 2, *F*_(2, 194)_ = 15.023, *p* < 0.001				0.030	0.028
Stressful life events at T1	0.166^*^	[0.027, 0.305]	2.360		

*N = 197. PSU, problematic smartphone use; In Step 2, only the newly included variable is presented. β, standardized coefficient beta; CI, confidence interval*.

* *p < 0.05*.

**Figure 3:**
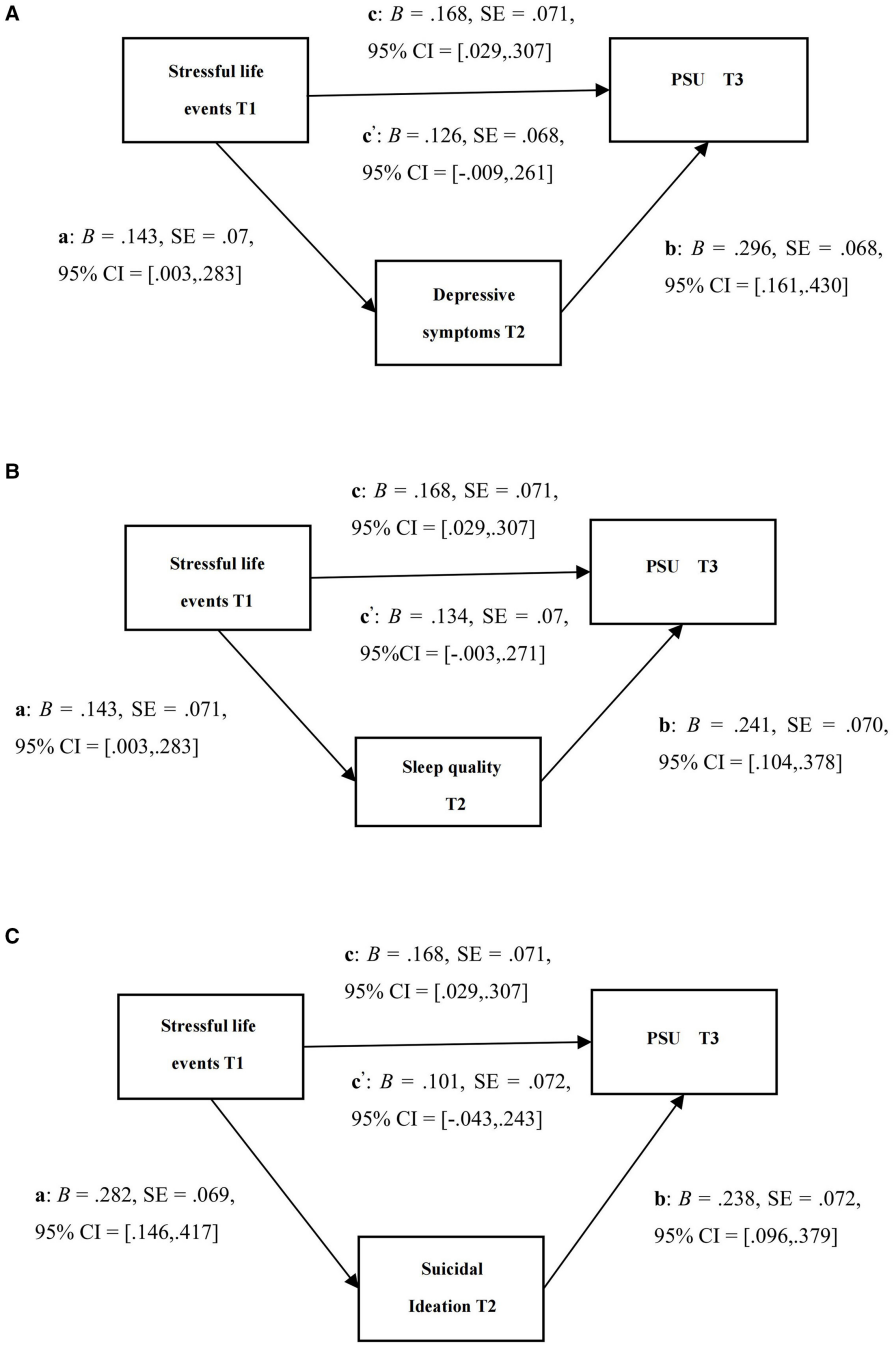
**(A)** The mediation model including stressful life events at T1, depressive system at T2, and PSU at T3; **(B)** The mediation model including stressful life events at T1, sleep quality at T2, and PSU at T3; **(C)** The mediation model including stressful life events at T1, suicidal ideation at T2, and PSU at T3. Note c: total effect, c′: direct effect; b: standardized regression coefficient, SE: standard error, CI: confidence interval.

Figure 3 shows the results of the mediation analyses. In the first model (Figure 3A), the total effect was significant (c: B = 0.168, *p* < 0.05), but the direct effect (c′: B = 0.126, *p* > 0.05) was not significant, which indicates that the depressive symptoms at T2 fully mediated the association between stressful life events at T1 and PSU at T3. The indirect effect (ab) was also significant (B = 0.042, 95% CI = [0.014, 0.098]; PM = 0.250); in the second model (Figure 3B), the total effect was significant (c: B = 0.168, *p* < 0.05), but the direct effect was not significant (c′: B = 0.134, *p* >0.05). This indicates that the sleep quality at T2 fully mediated the association between stressful life events at T1 and PSU at T3. The indirect effect (ab) was significant (B = 0.034, 95% CI = [0.003, 0.086]; PM = 0.202); In the third model (Figure 3C), the total effect was significant (c: B = 0.168, *p* < 0.05), but the direct effect was not significant (c′: B = 0.101, *p* > 0.05), which indicates that suicidal ideation at T2 fully mediated the association between stressful life events at T1 and PSU at T3. The indirect effect (ab) was significant (B = 0.067, 95% CI = [0.022, 0.124]; PM = 0.399).

## Discussion

The present three-wave longitudinal research was designed to examine the possible mediating roles of mental health problems (i.e., depressive symptoms, sleep quality, and suicidal ideation) at T2 in the linkage between stressful life events at T1 and PSU at T3 in a sample of Chinese undergraduate students. The current study is the first longitudinal study to highlight the significant mediating roles of mental health problems as a potential underlying mechanism that can explain the association between stressful life events and PSU. The results support our hypotheses.

We found that the levels of depressive symptoms at T1, T2, and T3 were not significantly different, while the levels of stressful life events, PSU, sleep quality, and suicidal ideation significantly changed over time. Exactly, the levels of stressful life events and PSU at T2 and T3 were significantly lower than that at T1, while the levels of sleep quality and suicidal ideation at T1 and T2 were significantly lower than that at T3. It is plausible that as students adjusted to college life and gained more access to different coping resources over time, they experienced fewer new stressful life events or were less likely to consider some events as stressful, and relied less on smartphones to cope with stress (70). Sleep quality decreased and suicidal ideation increased over time, which may be because of the pressures associated with graduation, job hunting, and lifestyle change as graduation approaches (71–73).

We found that stressful life events at T1 significantly predicted PSU at T3. Thus, Hypotheses 1 was supported. This finding corroborates the results of both cross-sectional (21, 74) and longitudinal studies (13, 23). Furthermore, our result is consistent with the I-PACE model (52). Stress is a hazardous trigger for problematic smartphone usage because it may reduce positive affect and impulse control and increase withdrawal in actual life, leading to passive stress coping, such as spending a great amount of time online to avoid real-life stress (75). Studying in universities, especially in the medical education environment is perceived to be stressful, and students may experience stressful events such as psychosocial and academic stress (76). In college students, the strong connection between stressful life events and problematic smartphone usage suggests that decreased stressful life events may decrease the risk of problematic smartphone usage. Smartphone addiction is not established in the study, but given the frequency of PSU amongst young people and its significant association with symptoms of common mental disorders (51), the potential causative factors of PSU require urgent further exploration.

The direct association between stressful life events at T1 and PSU at T3 was not significant when the mediator depressive symptoms were inserted into the linkage, revealing that depressive symptoms fully mediated this relationship. The result is consistent with the Diathesis Stress Model of Depression (33) and the I-PACE model (52), and corroborates the prior research (13, 77, 78). This finding suggests that stressful life events might exacerbate the level of depressive symptoms (34), and in turn increase PSU levels (14, 79). One possible reason is that stressful life events experienced by college students may lead to depressive symptoms (34), and individuals with depressive symptoms may rely on surfing the internet to alleviate their negative emotions (14). Furthermore, we found that sleep quality at T2 fully mediated the association between stressful life events at T1 and PSU at T3, which is consistent with “unsteady state” (40) and expanded the I-PACE model (52). That is, individuals who experience stressful life events are more likely to unable to fall asleep on time, their sleep problems are more serious (29, 41), such as lack of sleep and have difficulty in sleeping. In turn, people who have sleep problems are more likely to use mobile phones to kill time or seek to satisfy sleep problems caused by the tired, anxious, and daytime dysfunction of negative feelings, making individuals more willing to focus on mobile phones and increasing the time spent using mobile phones, thus leading to PSU (50). Suicidal ideation at T2 fully mediated the association between stressful life events at T1 and PSU at T3, which is consistent with the stress theory of suicide (44) and enriched the I-PACE model (52). That is, stressful life events may lead to suicidal ideation (30), and the anonymity of the Internet and the diversity of information can help them seek social support to alleviate or eliminate suicidal ideation (80), but misuse or overuse of smartphone may lead to PSU.

In general, the results suggests that mental health problems (i.e., depressive symptoms, sleep quality, and suicidal ideation) are important mediators in the relationship between stressful life events and PSU. Previous studies have shown that different types of stressful life events have different effects on mental health problems. For example, some studies have found that interpersonal stress and academic stress are associated with depressive symptoms, sleep quality, suicidal ideation, and PSU (81–83); another study showed that punishment significantly predicted depressive symptoms, but have no effects on poor sleep quality and suicidal ideation (84). Future research should examine the effects of specific types of stressful life events on different mental health problems.

### Implications

The results of this study have some important implications. First, these findings show that stressful life events may influence mental health problems in undergraduate students. Undergraduate students are faced with academic, lifestyle, and interpersonal pressures; thus, reducing the frequency and severity of stressful life events faced by undergraduate students early on could help to reduce future mental health problems (27, 29, 30, 34, 39). Second, by establishing mediation models, our findings can help practitioners understand the longitudinal relationship between stressful life events and PSU, and could aid the development of potential interventions. For example, implementing methods to enhance stress relaxation and adaptive stress coping skills, and increasing offline social support could help to reduce mental health problems and enhance sleep quality in undergraduate students, thereby reducing the negative effects of problematic smartphone use.

### Limitations and Further Research

The limitations of this study are as follows. First, it may not be conducive to the generality of the results of this study due to the convenience of sampling, and future studies could use random sampling methods to obtain more objective results. Second, the self-reported measures may be biased by self-evaluation, and further research could collect data from multiple resources (e.g., peers, parents, and teachers) to validate the present findings. Third, PSU and suicidal ideation were not assessed using diagnostic tools, future studies could test our models with clinical samples (such as smartphone addiction or Internet addiction). Fourth, different stressful life events may have different influences on undergraduate students' mental health problems. Future research should investigate the influences of different sorts of stressful life events on undergraduate students' mental health problems. Fifth, the reliabilities of the Pittsburgh Sleep Quality Index (0.68, 0.71, and 0.69) and the Self-Rating Idea of Suicide Scale (0.67, 0.72, and 0.71) were relatively low. Sixth, SEM was not used in this study to test the longitudinal relationships, which should be considered in future studies. Finally, measurement invariance is not taken into account when comparing mean values, which needs to be taken into account in future studies.

## Conclusion

The present study revealed that stressful life events and mental health problems (i.e., depressive symptoms, sleep quality, and suicidal ideation) are risk factors for PSU, and that mental health problems (i.e., depressive symptoms, sleep quality, and suicidal ideation) at T2 mediated the relationship between stressful life events at T1 and PSU at T3.

## Data Availability Statement

The datasets in the study are available from the corresponding author on reasonable request. Requests to access these datasets should be directed to zghcnu@wmu.edu.cn.

## Ethics Statement

The studies involving human participants were reviewed and approved by the Ethics Committee of the Wenzhou Medical University. The patients/participants provided their written informed consent to participate in this study.

## Author Contributions

XY and GZ prepared the study concept and design. CZ wrote the main manuscript text, analyzed the data, and edited the draft. HX, XL, XT, and ND conducted investigation and data curation. YL, DX, and GZ provided funds to conduct the study. All authors had full access to all data in the study, and take responsibility for the integrity of the data and the accuracy of the data analysis. All authors have approved the final version of the manuscript.

## Funding

This research was supported by the Social Science Foundation of China (BIA170166).

## Conflict of Interest

The authors declare that the research was conducted in the absence of any commercial or financial relationships that could be construed as a potential conflict of interest.

## Publisher's Note

All claims expressed in this article are solely those of the authors and do not necessarily represent those of their affiliated organizations, or those of the publisher, the editors and the reviewers. Any product that may be evaluated in this article, or claim that may be made by its manufacturer, is not guaranteed or endorsed by the publisher.
